# Remineralization Strategies in Oral Hygiene: A Position Paper of Italian Society of Oral Hygiene Sciences-S.I.S.I.O. Working Group

**DOI:** 10.2174/1874210601711010527

**Published:** 2017-10-24

**Authors:** Consuelo Sanavia, Marco Tatullo, Jessica Bassignani, Silvia Cotellessa, Giulia Fantozzi, Giovanna Acito, Alessia Iommiello, Lorella Chiavistelli, Silvia Sabatini, Gianna Maria Nardi

**Affiliations:** 1 Italian Society of Oral Hygiene Sciences-S.I.S.I.O. working group, Pisa, Italy; 2 Calabrodental SRL, Crotone, Italy; 3 Department of Oral and Maxillofacial Science, University of Rome “Sapienza”, Rome, Italy

**Keywords:** Dental Hygiene, Oral health, Dental Remineralizing, Enamel, Toothpaste, Mouthwash

## Abstract

**Background/Objective:**

The clinical conditions that lead to an alteration of the enamel structure are numerous. The diet high in sugars and acidifying substances, psychological stress that triggers parafunctional behaviors, the reduced intake of fiber-rich foods or alkalizing substances, together with other factors, contribute to demineralization of the tooth enamel. Dental mineralizing products on the current market are distinguished according to the dosage form, the active ingredient, the release technology, clinical indications and patient choice. Currently, it is necessary to propose to oral health professionals a guide to orient themselves in this chaotic choice, in order to prefer the most effective product for their own clinical target.

**Methods:**

Italian Society of Oral Hygiene Sciences-S.I.S.I.O. is one of the leading scientific Italian societies representing those dental hygienists working with high-quality standards and in agreement with scientific evidence: in the last year, the SISIO working group has carried out a study focused on remineralizing agents in dentistry, in order to give an authoritative point of view to indicate a guideline in the decision process of the choice of a remineralizing agent. We will report the results pointed out from the last consensus meeting in 2017.

**Results:**

We have reported the good the bad and the ugly have been discussed in a critical discussion of such topic.

**Conclusion:**

The SISIO experience has been reported in this position paper with the aim to serve as a useful aid in the daily choice of the clinical steps to perform, when dental professionals need to treat demineralized teeth.

## 1 INTRODUCTION

### 1.1 Dental Remineralizating Agents and Nanotechnologies

Dental caries is a chronic infectious disease resulting from the penetration of oral bacteria into the enamel and dentin. Microorganisms subsequently trigger inflammatory responses in the dental pulp [1]. Such disease is treated by means of invasive procedures aimed to remove the contaminated dental tissue, however, several strategies have been developed in order to give a new approach, such as the laser therapy [2].

The current literature suggests that demineralization should be retarded and remineralization should be enhanced to maintain a correct homeostasis able to protect the mineralized dental tissues [3].

However, this simple strategy seems to be unable to protect general population from carious lesions; in fact, caries is the most common dental disease worldwide [4].

A recent strategy is based on the treatment of early enamel lesions by innovative techniques and biomaterials [5].

Nanotechnology is constantly growing, and the applications of such technology on dentistry are daily increasing. Elkassas and Arafa [6] focused on the current status and the future implications of nanotechnology in preventive dentistry. Some nanomaterials showed antimicrobial effects making them a useful aid in the early-stage caries prevention, by intercepting early lesion progression, as nanosized calcium phosphate, carbonate hydroxyapatite nanocrystals, nano-amorphous calcium phosphate and nanoparticulate bioactive glass [6].

The challenge of clinicians is to achieve the best treatment of dental demineralization: this aim can be obtained with biomimetic biomaterials and innovative ways to carry them into the site of demineralization.

The repair of early dental demineralization has been recently achieved by peptides derived from milk caseins that associate with amorphous calcium phosphate (ACP) forming stable complexes.

### 1.2 *In-vitro* Studies: Casein Phosphopeptides Amorphous Calcium Phosphate

An *in-vitro* study [7] was performed on 100 specimens obtained from 50 human premolars to investigate the caries inhibitory effect of remineralizing agents and Er:YAG laser. Acid resistance of human enamel was investigated with *in-vitro* experiments.

The used products were made from Casein Phosphopeptides Amorphous Calcium Phosphate (CPP-ACP) [GC Tooth mousse] cream and Casein Phosphopeptide Amorphous Calcium Fluoride Phosphate (CPP-ACFP) [GC Tooth mousse plus] cream; on the other hands, the laser utilized was Er:YAG laser alone, and also a combination of CPP-ACP with Er:YAG laser, and CPP-ACFP with Er:YAG laser.

The lowest mean score of calcium loss, after acid attack with some acid substances typically used in such kind of experiments, was observed for CPP-ACFP with Er:YAG laser followed by CPP-ACFP but the differences between these groups were statistically not significant (*p*>0.05).

This study suggested that the combination of CPP-ACFP with Er:YAG laser is more effective in decreasing enamel demineralization, under acid conditions, when compared with other groups.

### 1.3 *In-vitro* Studies: Bioglass-phosphoric Acid

Since the products aimed to remineralize are numerous and each of them is based on a specific mechanism of action, it is interesting to consider that the literature has reported studies on almost all the active principles now present in the market. El-Wassefy *et al*. [8] investigated the combining effect of cold plasma and bioglass-phosphoric acid paste on demineralized enamel. Specimens were divided into five groups: (I) Control, demineralized enamel (C); (II) Demineralized enamel treated with fluoride varnish (F); (III) Cold plasma application to demineralized enamel (P); (IV) Demineralized enamel treated with bioglass paste (B); (V) Application of bioglass paste to cold plasma-treated demineralized enamel (PB). After having placed all specimens in remineralizing solutions for 24 h, they were accurately examined; the results showed that treating demineralized enamel with cold plasmas before the bioglass application ensured a significantly higher mineral volume recovery and micro-hardness of demineralized region, thus, shedding an interesting light on the role of cold plasmas as important playmaker of the correct remineralization of demineralized enamel.

### 1.4 *In-vitro* Studies: Tri-calcium Phosphate (TCP) and Fluoride (F)

Many biological processes are supported or promoted by inflammatory conditions, and many biological environments suffer from the impairment of oxidative stress levels [9-11].

Polyphenols have been demonstrated to be effective against several forms of oxidative stress, and in some cases, they have been also reported to be an *in-vitro* anticancer agents [12].

Proanthocyanidin is a well know polyphenol, and it has been shown to enhance dentine collagen stability and remineralization of artificial root caries. Epasinghe *et al.* [13] evaluated the effect of proanthocyanidin (PA) in combination with tri-calcium phosphate (TCP) and fluoride (F) on resistance to collagen degradation and remineralization of artificial caries lesions. Results showed that the lowest lesion depth and mineral loss were observed in the TCP+F+PA (*p*<0.05) group. The addition of PA to TCP+F also decreased hydroxyproline release, so the addition of PA to TCP+F reduced collagen degradation, inhibited demineralization and enhanced remineralization. This result clearly shows how tri-calcium phosphate and fluoride are the basis of the mineralizing process, but also other biological agents can have an impact on dental remineralization.

Majithia *et al.* [14] compared and evaluated the remineralization potential of three commercially available varnishes on artificial enamel lesions. Based on their results, all the three commercially available varnishes were capable of remineralizing initial enamel lesions that were induced artificially. No difference was noted in the remineralizing efficacy of the varnishes despite their different compositions. Varnish based on casein phosphopeptide-amorphous calcium phosphate fluoride showed slightly better recovery in surface micro-hardness as compared to the other varnishes. All the varnishes used in this study were however able to reverse the early-stage enamel lesions.

### 1.5 *In-vitro* Studies: Sodium Hexametaphosphate (HMP)

Sodium hexametaphosphate (HMP) is an inorganic salt and its functions can be related to the role of chelating agent as well as of corrosion inhibitor. da Camara *et al*. [15] evaluated the effect of fluoride dentifrices combined with sodium hexametaphosphate (HMP) on enamel demineralization *in-vitro*. The study was performed on enamel bovine blocks and a placebo without fluoride and without HMP was also investigated. HMP1% promoted the lowest mineral loss, and led to significantly lower demineralization in the deeper regions of the subsurface lesion when compared with the other HMP-containing toothpastes. These results showed how the supplementation of an 1100-ppm F dentifrice with 1% HMP promoted a higher inhibitory effect against enamel demineralization when compared to a dentifrice containing the same amount of fluoride *in-vitro*, and it is indicated to patients at high risk of caries.

### 1.6 *In-Vitro* Studies: Xylitol

In the recent time, the dysmetabolic pathologies such as diabetes have put great attention on diet and on the composition of many oral health products. Carbohydrates are usually not suggested in a specific diet, in this light, many products have been tried to use other sugars such as xylitol.

Cardoso *et al*. [16] analyzed the effect of varnishes containing xylitol compared to commercial fluoridated varnishes on the remineralization of artificial enamel caries lesions *in situ*. In this research, artificial caries lesions were created and each specimen in each subject were treated once with different varnishes: 20% xylitol varnish seemed to be a promising alternative to increase surface and subsurface remineralization of artificial caries lesions *in situ*.

### 1.7 *In-Vitro* Studies: Xylitol

Bioactive glasses are a group of surface reactive glass-ceramic biomaterials. They can be considered as an alternative to nearly inert implant materials, and they are commonly used in dentistry. Palaniswamy *et al.* [17] evaluated remineralizing potential of bioactive glasses (BAGs), using as control the amorphous calcium phosphate-casein phosphopeptide (ACP-CPP) on superficial enamel lesions. The samples showed a reduction of the microhardness after demineralization; on the other hands, after application of remineralizing agents, both the tested remineralizing agents tested in this study have been effective in repair and prevention of demineralization. BAG showed better results initially, but in the late stages, both BAGs and ACP-CPP showed to have similar remineralizing potential.

### 1.8 *In-Vitro* Studies: Stannous Fluoride (SnF2)

Dental market is rich in product based on fluoride: the most used fluoride sources today are sodium fluoride and stannous fluoride: the first is able to kill bacteria, however, both have been shown to rebuild enamel. In fact, both types of fluoride can prevent tooth decay by protecting tooth enamel against acid erosion caused by bacteria.

Gangrade *et al*. [18] tested the efficacy of stannous fluoride (SnF2), in comparison with casein phosphopeptide-amorphous calcium phosphate with fluoride (CPP-ACPF) and calcium sucrose phosphate (CaSP). After a superficial demineralization of the enamel, a remineralization procedure was carried out by using SnF2, CPP-ACPF, and CaSP. The results showed that the 3 tested remineralizing agents improved surface remineralization, but all ensured the complete process after the deadline of 7 days. SnF2 showed the highest potential for remineralization followed by CaSP and CPP-ACPF, highlighting the important role of this salt in the dental protection.

Many dental products have some compounds that could interact with the surface of dental appliances, as happens with dental implants as well as with the orthodontic brackets: the knowledge of also such behavior can affect the choice of the mouthwashes in case of remineralizing therapy. An Italian study reported the modification of the surface roughness induced by treatment with mouthwash containing amine fluoride and another containing zinc-substituted carbonate–hydroxyapatite. The treatment with fluoride based mouthwash showed a deep roughness in the streaks on the titanium bracket surface; this roughness was reported also in the metallic dental implants, causing bacterial contamination leading to severe and early infections in the implant site. On the contrary, the *in-vitro* treatment with a mouthwash containing zinc-substituted carbonate–hydroxyapatite reduced the surface roughness, inhibiting the bacterial growth on the implant-supported prostheses. These results should be taken into consideration when we suggest a treatment based on mouthwashes containing amine fluoride [19].

Commonly, the remineralization of a superficial layer of dental enamel is not easy to control, to achieve both a good aesthetic and functional result. The regeneration of the enamel lesions by promotion of a new mineralization in the most biomimetic pathway to ensure the formation of dental enamel, mimicking the natural biomineralization approach for enamel remineralization. Wang *et al*. [20] simulated the biological process of enamel development proteins, such as amelogenin. This study achieved a biomimetic remineralization process through the oriented attachment (OA) of nanoparticles based on non-classical crystallization theory. The interesting results indicates that the use of ECM proteins such as amelogenin in the biomineralization process, thus imitating the process of biomineralization, would be a successful strategy for enamel remineralization.

Apart from the additive strategies of remineralization Kerr *et al.[* 21] developed a novel approach for noninvasive treatment of dental caries based on killing *Streptococcus mutans* with high-frequency microwave energy (ME). In fact, the modulation the pH of carious lesions is able to stimulate the spontaneous tooth remineralization. Microwaves can kill the vast majority of S. *mutans in-vitro*, and this killing action made on S. *mutans* by ME promotes effective remineralization of S. *mutans*-demineralized enamel. Also this last approach should be taken into consideration for the management of early caries in minimal invasive dentistry (MID).

## 2 METHODS

Italian Society of Oral Hygiene Sciences-S.I.S.I.O. is one of the leading scientific Italian society representing those dental hygienists working with high-quality standards and in agreement with scientific evidence: in the last year, the SISIO working group has carried out a study focused on remineralizing agents in dentistry, in order to give an authoritative point of view to indicate a guideline in the decision process of the choice of a remineralizing agent. (Table **1**).

We will report the results pointed out from the last consensus meeting in 2017.

In our experience, we have analysed several products, each of them showed characteristics and advantages that we feel useful to briefly describe.

## 3 RESULTS

Some products are based on Stannous Fluoride which demonstrated the ability to dissociate into fluorine ion (anti-caries) and stannous ion (multi-therapeutic): the latter is unstable in water, losing nearly all its therapeutic capacity, because it easily forms the hydroxide. For this reason, Stannous Fluoride has been stabilized with two strategies: 1) formulation with a very low water content, 2) in the presence of chelating agents such as gluconate that are capable of sequestering the stannous ion, protecting it from hydrolysis and oxidation, but able to subsequently release the ion, therapeutically active on the surface of teeth and mucous membranes. Instead, the combination of sodium fluoride and calcium and the formation of calcium fluoride deposits, facilitate the conversion of long-term hydroxyapatite into fluorapatite, contributing to remineralization.

### 3.1 Role of Morphology of Carriers and of Releasing Strategy

Another interesting product contains *microbiorepair particles,* small particles consisting of hydroxyapatite whose composition is very similar to that of the enamel. This similarity gives to microrepairs some biomimetic properties that allow the microparticles to integrate with enamel and dentin resulting in a mineralizing activity. This strategy allows to avoid to develop aggressive carious lesions, reducing the risk of pulp pathologies potentially affecting also systemic conditions [22-24].

The new technologies have allowed to create new products/formulations, often patented, as the fTCP - functionalized tricalcium phosphate- which ensures optimal and controlled mineralization due to the release of specific doses of calcium. It consists of crystalline Beta-tricalcium phosphate, a natural precursor of hydroxyapatite, thus mimics the biological mineralization process. Mineralize at a superficial and subsurface level; it operates in synergy with the saliva and fluoride. The saliva activates the fTCP and starts the calcium release process. The calcium and phosphate, released in low concentrations, act as a base to start the mineralization process in a controlled manner, mimicking the natural process of biomineralization. So fTCP and fluorine act synergistically to form a better quality mineral crystals, relatively large and densely compact. The fluorine accelerates the mineral growth, fTCP acts as a reserve for supplying the biological remineralization process.

The remineralization is a process relative to the hard tissues: these mineralized tissues are naturally produced by specific cells derived by the stem cells residing in dental pulp after differentiation processes [25-27].

The classic, but ever working, sodium fluoride varnish provides a high fluoride absorption and a proven occlusion of dentinal tubules, thus, it reduces the dentinal hypersensitivity in addition to the main function of remineralization of enamel. The alternative to the previous product is based on Fluoride silane; it dissolved in aqueous solution of ethyl acetate and isoamyl propionate, substances that facilitate the diffusion of fluoride ions and the adhesion to the enamel. The innovative releasing-system, thanks to its content homogeneously dissolved fluoride, allows the immediate availability of this component. Fluor Protector releases in a short time the entire contents of fluorine, thus it is guaranteed a direct and effective fluorination of the dental enamel. After the application of this product, finely distributed particles of calcium fluoride are carried on teeth. The thick cover layer acts as a storage, from which fluorine and calcium are released with prolonged action over time. As a result, the acids cannot directly attack enamel and dentin.

### 3.2 Role of Chemical Factors in Controlling the Enamel Lesions and Dentinal Sensibility

Many toothpastes work in several ways, such as by regulating the changes in the pH of the plaque, by preventing the adhesion and growth of *Streptococcus mutans* and *Streptococcus sobrinus* on the tooth surface, by remineralizing the enamel lesions and makes it more resistant to acid attacks and by optimizing the absorption and transport of fluoride on the enamel.

Some of them contain the CPP-ACP (fosfopeptidecaseino-amorphous calcium phosphate) a derivative of milk casein. The CPP-ACP complex is able to fight against acid attacks and carry calcium and phosphate ions on the enamel in a highly absorbable form.

This mechanism ensures the contrast of demineralization and at the same time the remineralization of the tooth structure making it more resistant.

Remineralizing products are made by different and numerous compounds, some of them are biomaterials used as scaffold or fillers [28-30]. Many of them are quite often present in the common formulations, thus, we think it’s interesting to briefly report their characteristics.

Carbonate promotes the release of calcium and phosphate ions from hydroxyapatite, as well as the release of other ions substituents magnesium, strontium and fluoride. The carbonate ion is present in abundance in the hydroxyapatite during bone formation, and then decrease in mature bone. The carbonate greatly reduces the stability of HA, raising its solubility in physiological environment and its ability to release calcium and phosphate ions [22-25].

Strontium desensitizing effect and synergistic action with fluoride has remineralization effects of enamel; the combination fluoro-strontium determines a higher antibacterial action. The incorporation of strontium in crystals in mineral phase promotes its stabilization, preventing further sensitivity phenomena. Strontium has a stimulating function of the bone formation process, for this is found in many drugs as anti-accelerator of the osteoporotic process [25].

Magnesium accelerates the nuclearization process of new crystals of hydroxyapatite and slows it volumetric growth. It will be generated a considerable number of small, more compact crystals with an increased specific surface area with high bioactivity. The magnesium substituent calcium in HA molecule, confers remineralization action, useful both in the formation of new enamel crystals and to promote the regeneration of the dentin; we can also consider as interesting the role of Chitosan, as it can improve the characteristics of muco-adhesion to enamel, so we could use a combined strategy with such materials.

Xylitol is a naturally occurring polyalcohol has anti-caries and anti-bacterial properties; it is absorbed as a sugar but it is not converted into cariogenic metabolites such as lactic acid [16].

Fluoride modifies the crystalline structure of biological hydroxyapatite and stabilizes it decreasing its solubility and reactivity. In bio-enamel the fluorine is administered as fluoro-hydroxyapatite form that allows the release of active components. The hydroxide ions present in the pseudo-hexagonal crystalline lattices, are directly exposed on the interprismatic surface thus they are strongly attackable by the H + ions of oral liquids. Their replacement with fluorine ions strongly reduces the solubility of hydroxyapatite transforming it into fluoro-hydroxyapatite with monoclinic crystal lattice and therefore more stable to acid attack [7, 17, 18].

Formulation is an important element of choice that could affect the efficacy of the product. Nowadays, many products are delivered in gel-formulation. An example is a gel composed of di-hydro-fluoride of bis-amino-propyl-N- hydrossiel-octadecylamine in addiction with flavouring agents and hydroxyethyl cellulose. Gel is considered to have a better action, given its ability to work in the areas difficult to reach by a common paste-formulation [17]. Thanks to the topical action of fluorides, as it affects hard tissues of the tooth at 2 levels: the first is the increasing of enamel resistance to the corrosive action of fermentation products, another level of action is on the inhibition of the growth and development of the bacteria responsible for tooth decay. So, the strategy is to act on hard tissues, even if, in the general management of oral health, also soft tissues should be protected and regenerated [31-33].

### 3.3 Biomimetic Strategies

Usually, remineralizing products are formulated in order to ensure a dos/release activity: in fact, the product, absorbed by the enamel, is stored and released slowly over time.

Many products are following the general criteria of the regenerative medicine in their action. One of the most important concept is the “biomimetism”, the natural ability of a compound to mimicking the physiologic activity of a tissue [34]. The use of crystals of synthetic hydroxyapatite (carbonate-hydroxyapatite-zinc-acting) ensure to reproduce a “biomimetic” behaviour very similar to the natural component of the tooth. Products containing this formulation are capable of repairing the enamel of the teeth thanks to the special molecular structure of the crystals of synthetic hydroxyapatite. This molecule has a mineralizing action as it adheres in a biomimetic manner to the tooth surface, with a repairing effect that lasts over time [35].

The last frontiers of dental healthcare are related to the use of new technologies to deliver the products, or to improve their effects on the surface of teeth (Table **2**).

In such cases, the products are delivered by the iontophoresis, in fact, this technology has the advantage of bringing an adequate amount of drug in a specific area, improving the topical action of the formulation used. [36]

Furthermore, UV-rays are easy to use and useful to start some chemical reactions: varnishes are often applied in liquid state, but they are able to be hardening after being undergone to UV rays; moreover, UV and photothermal lamps have bactericidal action, leaving intact the tooth structures. An example is represented by the photodynamic therapy: it is able to destroy 99% of the bacteria present in the bacterial biofilm.

The ozone therapy is another useful and widely used technology: this therapy, if applied to the carious tooth surface, is able to obtain an effective regression of caries, because it is able to stop the growth of bacteria and eliminate them progressively [37].

These technologies are useful to improve the products activity but we can use them also in clinical diseases, such as gingivitis, pericoronitis or periimplantitis.

## CONCLUSION

Italian Society of Oral Hygiene Sciences-S.I.S.I.O. involve the vast majority of dental hygienists working in private and public healthcare centers. The main scope of SISIO is to improve the current guidelines regarding the most timely topics related to oral health, within the limitations of the professional profile of dental hygienist. The SISIO-working group has reported in this position paper the experience reported by a large group of professionals representing the whole SISIO, in order to give a clear point of view and a useful guideline when dental professionals need to treat demineralized teeth.

A concrete aid to reduce/prevent the demineralization processes is represented by products mainly based on stabilized Stannous Fluoride which have revealed therapeutic activities both on the teeth surfaces, thanks to the formation of calcium fluoride deposits that promote the formation of fluorapatite, and on the soft tissues.

The different active drugs contained into the main oral remineralizing products could also be strongly carried on active sites by means of carriers able to influence the releasing features of the products. Microparticles are very promising in such field, since these carriers are able to treat and fill the enamel lesions.

Also the role of chemical agents in controlling the enamel lesions and dentinal sensibility have been properly reported; the current strategy is to regulate the pH by means of Chemical compounds that act through an effective buffer effect: some of them are particularly interesting and they are based on the CPP-ACP (fosfopeptidecaseino-amorphous calcium phosphate) a derivative of milk casein; nevertheless, also the traditional and well assessed molecules, such as the xylitol, have been confirmed as useful and highly preventive against the caries formation, especially in the youngest patients.

The future insights look with great interest towards the biomimetic products, able to be highly-targeted and with the ability to diagnose and treat the main enamel defects; iontophoresis and ozone therapy are also useful to maximize the therapeutic effects of such new products.

The future seems to be right now, we only need to correctly use the new products inside our traditional treatment plane: we hope this position paper will stimulate the readers to enlarge their own limits and to actively try new therapeutic approaches, in order to eradicate the severe forms of teeth demineralization.

## Figures and Tables

**Table 1:** The following table lists the names, the company, the active substances, properties, formulations of the products investigated by SISIO working group.

**Name**	**Company**	**Active Substance(s)**	**Type of Action**	**Formulation**	**Main Advantages**
**Az pro-expert rigenera smalto**	Procter & gamble	Stabilized stannous fluoride	Remineralisation and protection of the enamel	Toothpaste	Good persistence on teeth surfaces.
**Biorepair**	Coswell	Hydroxyapatite (formulation biorepair® zincoPCA®)	Repair and regeneration of the enamel, antibacterial	Toothpaste	Good action on teeth sensibility.
**Clinpro™ white varnish**	3M ESPE	Sodium fluoride 5% Tri-Calcium phosphate (fTCP)	Remineralisation desensitization prevents caries formation (fTCP 3M ESPE innovative technology)–	Vernice	Good action on teeth sensibility.
**Desensitising mousse**	Henry schein	Potassium nitrate 4,2% Xylitol 30% sodium fluoride 1450ppm	Remineralisation	Mousse	Good persistence on teeth surfaces.
**Duraphat fluoride varnish**	Colgate	Sodium fluoride 22,600ppm	Remineralisation	Varnish	Good action on teeth sensibility.
**Elmex protezione erosione**	Gaba	Stannous cloride 800ppm chitosan 0,5% amine fluoride 700ppm sodium fluoride 700ppm	Remineralisation prevention of dental erosion	Toothpaste	Good action on soft and hard dental tissues.
**Enamelast**	Ultradent	Sodium fluoride 5% xylitol	Remineralisation	Varnish	Flavour appreciated by patients
**Exense**	Cavex	Hydroxyapatite	Remineralisation	Cream	Good persistence on teeth surfaces.
**FlourProtector**	Ivoclar vivadent	Fluoride 0,1% silane	Remineralisation	Varnish	Good action on teeth sensibility.
**Medical Gel di fluoro neutro**	Medical	Amine fluoride, sodium monofluorphosphate	Remineralisation	Gel	Good protection against acid substances
**MI Varnish**	GC	Fluorine 22.600ppm CPP-ACP (phosphopeptide casein -amorphous calcium phosphate), recaldent® 2%	Remineralisation	Vernice	Good action on teeth sensibility.
**Mi paste plus**	GC	ACP(F) + sodium fluoride 900ppm	Remineralisation antibacterial	Cream	Good persistence on teeth surfaces.
**Regenerate enamel science**	Unilever	Sodium monophosphatefluoride 1450ppm e NR-5™ – calcium silicate and sodium phosphate	Remineralisation	Toothpaste	Flavour appreciated by patients
**Remin Pro**	Voco	Hydroxyapatite fluoride 1450ppm xylitol	Remineralisation	Cream	Good persistence on teeth surfaces.
**Sensout**	Ids	Amine fluoride nano calcium fluoride nano fluorapatite citrate zinc potassium chloride	Remineralisation Promote developing of secondary dentine	Toothpaste	Flavour appreciated by patients
**Stomyprox**	Croswell	Hydroxyapatite carbonate Zinc RDA 27	Remineralisation antibacterial protection	Polishing paste	Good antibacterial activity.
**Tooth mousse**	GC	CPP-ACP (fosfopeptide casein amorphous calcium phosphate), Recaldent®	Remineralisation Antibacterial	Cream	Good persistence on teeth surfaces.
**Clinpro™ white varnish**	3M ESPE	TCP (Tri-Calcium phosphate), fluoride 22,660 pm	Remineralisation desensitization.	Vernice	Good action on teeth sensibility.
**Biorepair**	Croswell	Hydroxyapatyte biorepair® zinc PCA®	enamel microrepair	Mouthwash	Good protection against acid substances
**Biosmalto**	Curasept	hydroxyapatite chitosan fluoride	Remineralisation caries prevention	Toothpaste	Flavour appreciated by patients
**Elmex gel**	Colgate palmolive	Olaflur sodium fluoride dectaflur fluoride 1,25%	Remineralisation caries prevention	Gel 25gr	Flavour appreciated by patients
**Elmex gel dentale**	Colgate palmolive	Olaflur sodium fluoride dectaflur fluoride 1,23%	Remineralisation caries prevention	Gel 215gr	Flavour appreciated by patients
**Elmex protezione carie**	Gaba	Amine fluoride 250ppm	Remineralisation enamel strengthening	Mouthwash	Good action on soft and hard dental tissues.
**Elmex protezione erosione**	Gaba	stannous chloride amine fluoride Sodium fluoride 500ppm	Remineralisation	Mouthwash	Good action on soft and hard dental tissues.
**Flairesse**	Dentalica	Fluoro xilitolo	Remineralisation	Gel/foam	Good persistence on teeth surfaces.
**Fluor protector gel**	Ivoclar vivadent	Fluoride 1450ppm calcium phosphate xylitol	Remineralisation antibacterial	Gel	Good protection against acid substances
**Medical gel** **acidulated fluoride**	Medical	Acidulated sodium fluoride 0,33%	Remineralisation	Gel	Good protection against acid substances
**Rigenerate enamel science**	Unilever	NR-5™ – calcium silicate and sodium phosphate	Remineralisation enamel strengthening	Serum	Protection from bacterial contamination.
**Sensivital gum**	Sunstar	Fluoride 250ppm	enamel strengthening	Mouthwash	Good protection against acid substances

**Table 2:** Mineralizing associated technologies.

**Technology**	**Description**	**Application**	**Action**	**Main Advantages**	**Company**
**Iontophoresis**	The iontophoresis or iontophoresis (ion-phòresis = ion transport) is a pharmaceutical administration technique that uses a direct current (galvanic current), produced by a suitable generator for topical administration of a drug in the ionic state	Optimal topical action, avoiding systemic administration and introducing the only active ingredient without excipients	Transport of substances	Iontophoresis has the advantage of bringing an adequate amount of drug in a localized area.	Jonofluor (MEDICAL)
**Photo-polymerization**	The polymerization reaction starts and is supported when the photosensitive agent is maintained in its excited state; these make it reactive with the reducing agent to produce free radicals, which open the double bonds C = C of the methacrylate groups making bonds between them (polymerization)	It is possible, for certain applications, to formulate and to produce varnish hardening with UV rays	Substance fixing	Local action, easy to be used also by not-expert dental hygienists and dentists.	Valo (ULTRADENT)
**Diode laser**	It is produced a beam of light generated as amplification of a photon input that stimulate the emission.	Photothermal type bactericidal action, it can destroy bacterial cells while leaving intact the tooth structures by exploiting the photodynamic therapy	Decontamination	Multiple actions: decontamination of the site and photo-dynamic therapy.	Helbo (BREDENT)
**LED**	The therapeutic protocol is based on the use of a photosensitive substance, Toluidine Blue (TBO), which is activated by a light source at a given wavelength. The received energy emanates reactive oxygen with cytotoxic effect on the cell membrane of microorganisms	It is able to destroy 99% of the bacteria present in the biofilm, even those resistant and / or susceptible to antibiotics. The treatment can be repeated several times without causing side effects and the bactericidal effect is immediate. The major applications include gingivitis and pericoronitis, the perimplantitis, the treatment of periodontal pockets and endodontic disinfection.	Decontamination	Optimal antibacterial action: useful in acute inflammatory conditions.	Fotosan (DENTALICA)
**Ozone**	Ozone is one of the most effective oxidant, destroys bacteria, viruses or fungi, and carries out a hemostatic action. The ozone produced is delivered at the precise point to be treated.	The ozone applied to the carious tooth surface is able to obtain an effective regression of caries, because it is able to stop the growth of bacteria and eliminate them progressively. Not only it stops caries, but the diseased substance, preserved and subjected to ozone, is remineralises and hardens within 4-12 weeks	Decontamination	Useful in conditions with flogosis substained by bacteria. Useful to avoid an invasive procedure by dentists.	HealOzone (KAVO)
